# Therapeutic interventions targeting enteropathy in severe acute malnutrition modulate systemic and vascular inflammation and epithelial regeneration

**DOI:** 10.1016/j.ebiom.2024.105478

**Published:** 2024-12-10

**Authors:** Jonathan P. Sturgeon, Kuda Mutasa, Mutsa Bwakura-Dangarembizi, Beatrice Amadi, Deophine Ngosa, Anesu Dzikiti, Kanta Chandwe, Ellen Besa, Batsirai Mutasa, Simon H. Murch, Susan Hill, Raymond J. Playford, Kelley VanBuskirk, Paul Kelly, Andrew J. Prendergast, the TAME Trial Team

**Affiliations:** a Zvitambo Institute for Maternal and Child Health Research, Harare, Zimbabwe; b Blizard Institute, Queen Mary University of London, London, UK; c Tropical Gastroenterology and Nutrition Group, University of Zambia, Lusaka, Zambia; d Faculty of Medicine and Health Sciences, University of Zimbabwe, Harare, Zimbabwe; e Warwick University Medical School, Coventry, UK; f Great Ormond Street Hospital, London, UK; g University of West London, London, UK; h University College Cork, Ireland

**Keywords:** Severe malnutrition, SAM, Severe acute malnutrition, Systemic inflammation, Vascular inflammation, Randomised trial

## Abstract

**Background:**

Severe acute malnutrition (SAM) is the most life-threatening form of undernutrition, and children hospitalised with complications have unacceptably high mortality. Complicated SAM is a multisystem disease characterised pathophysiologically by muscle wasting, systemic inflammation, metabolic dysfunction, and malnutrition enteropathy including epithelial barrier dysfunction. There is a clear need for novel interventions to address the underlying pathogenic perturbations of complicated SAM.

**Methods:**

In this analysis of tertiary outcomes from a phase II multi-centre trial in Zambia and Zimbabwe, multiplex biomarkers were measured in 122 children (57% male) with SAM randomised following stabilisation (‘baseline’) to one of four interventions for 14 days to treat malnutrition enteropathy: budesonide, N-acetylglucosamine, colostrum, or teduglutide, compared with standard-of-care. Following measurement of 35 biomarkers from day 15 plasma samples using Luminex and ELISA, the dimensionality of biomarker data was reduced using principal component analysis.

**Findings:**

Both budesonide and colostrum reduced systemic inflammation (as measured by CD14, IL1-ra, CRP, and LBP), while children receiving colostrum had higher GLP2 and angiopoietin, and lower circulating lipopolysaccharide, suggesting better restoration of epithelial barrier function. N-acetylglucosamine, a precursor for epithelial glycosaminoglycan synthesis, increased biomarkers of epithelial regeneration (EGF, VEGF), and circulating growth factors (angiopoietin, IGFBP-3, and GCSF).

**Interpretation:**

Interventions aimed at ameliorating malnutrition enteropathy showed plausible effects on biomarkers of inflammation and epithelial regeneration, demonstrating an interdependence of systemic inflammation and enteropathy markers seen in structural analysis. Given the interplay between inflammation and tissue restoration in malnutrition, this mechanism of action supports larger trials to determine the clinical benefits of interventions, either alone or in combination, in children with complicated SAM.

**Funding:**

This analysis of tertiary outcomes for the TAME trial was funded by a Wellcome grant to JPS (220566/Z/20/Z). The TAME trial was funded by a grant from the Medical Research Council (UK), number MR/P024033/1. AJP is funded by Wellcome (108065/Z/15/Z). Takeda UK provided teduglutide at a discounted price.

## Introduction

Severe acute malnutrition (SAM) is the most life-threatening form of undernutrition, estimated to affect 13.7 million children under five years old in 2022.^1^ The World Health Organization (WHO) defines SAM in children 6–59 months old as a weight-for-height z-score <−3, mid-upper arm circumference of <115 mm, or bilateral pitting oedema.^2^ Although most children with SAM receive community-based management, those with complications such as poor appetite, bilateral severe oedema, signs of metabolic disturbance or infections, or danger signs such as persistent vomiting or seizures, are admitted to hospital. The inpatient mortality of children with complicated SAM in a recent meta-analysis was 15.7% (range 3.7%–41.4%) across 19 studies largely from sub-Saharan Africa.^3^ Mortality also remains high after discharge, with one Zimbabwe/Zambian study from the same centres as this trial showing the one-year post-discharge mortality^4^ almost matching the inpatient mortality, and being replicated in other studies across sub-Saharan Africa.^5^ There is a clear need for transformative therapeutic approaches to improve outcomes.

Complicated SAM manifests as a multisystem disease, which includes sarcopenia and/or oedema, reduced intestinal villus height and crypt hyperplasia,^6^ metabolic disturbance, skin lesions likely due to extracellular matrix dysfunction^7^ in oedematous SAM, and immune changes consistent with a functional immunodeficiency. A recent narrative review on this topic by us and others in the field has hypothesised that inflammation underlies many of these changes,^8^ with inflammation being a cause and/or consequence of these changes. Children hospitalised with SAM have high inflammatory biomarkers from across several studies and settings, but since many children present with infection, pathogen-specific immune responses likely contribute. However, changes appear to persist over one-year post-discharge,^9^ suggesting infection is not the sole cause of the chronic inflammation apparent in children with malnutrition. There appears to be an interplay between inflammation and growth/regeneration, with biomarkers reflecting these competing processes negatively associated with each other, and independently predictive of mortality.^9^ Higher systemic inflammatory biomarker concentrations are independently predictive of mortality in children with SAM,^10–12^ and associated with poorer linear growth in the 6 months following discharge.^13^ Chronic inflammation has been shown to alter monocyte phenotypes,^14^ and likely to induce a state of functional immunosuppression, leaving children vulnerable to further infections,^15,16^ and to impaired tissue growth and repair. Currently, treatment of SAM does not adequately address this multi-organ dysfunction and inflammation.

Therapeutic Approaches to Malnutrition Enteropathy (TAME) was a Phase II trial conducted among children admitted with complicated SAM in Zimbabwe and Zambia. Four interventions aimed at improving malnutrition enteropathy were initiated after clinical stabilisation: budesonide, colostrum, N-acetylglucosamine, or teduglutide. These were selected due to their 4 distinct modes of action: 1) oral budesonide reduces local inflammation, 2) colostrum aimed to reduce epithelial permeability, 3) N-acetylglucosamine is a sugar needed for epithelial regeneration, and 4) teduglutide, a glucagon-like peptide-2 (GLP2) analogue, promotes growth and repair in the intestine. Teduglutide showed an improvement in the trial primary outcome—an enteropathy score comprising faecal biomarkers of intestinal inflammation. Here, we progress to examine the tertiary endpoints of the TAME trial, evaluating multiple plasma biomarkers using a multiplex platform. Building on our findings, we now explore whether the randomised interventions improve the interplay between systemic/vascular inflammation and markers of intestinal repair and growth, which are known pathophysiological pathways present in complicated SAM.^9^

## Methods

The TAME trial investigated the impact of four interventions on enteropathy biomarkers in a multi-arm, phase II, randomised controlled trial in southern Africa between 4th May 2020 and 27th April 2021, when the trial recruited the required number of children. The protocol has been published.^17^ Briefly, 125 children hospitalised with SAM were randomised to standard of care, or one of four investigational medicinal products (IMPs) for 14 days: oral budesonide, oral bovine colostrum, oral N-acetylglucosamine, or subcutaneous teduglutide.^17^ Randomisation and interventions were given following stabilisation of the child, which constituted the trial baseline. TAME was conducted at two tertiary referral hospitals in Harare, Zimbabwe (Sally Mugabe Hospital), and Lusaka, Zambia (Children’s Hospital - University Teaching Hospital).

### Endpoints of the trial

The primary endpoint of the TAME trial was the change in a composite enteropathy biomarker score^17^ derived from Kosek et al., incorporating faecal alpha-1-antitrypsin, neopterin, and myeloperoxidase^18^ between baseline and day 15. Only teduglutide improved the composite primary outcome.^19^ The current analysis evaluated tertiary endpoints included in the trial protocol, which were designed to explore changes between baseline and day 15 in 16 circulating biomarkers of systemic inflammation, 6 biomarkers of endothelial activation, and 7 circulating growth factors. A full list of the soluble plasma biomarkers analysed is shown in Supplementary Table S1.

### Recruitment and randomisation

Inclusion criteria were children aged 6–59 months hospitalised with SAM according to WHO criteria. Children were recruited after initiation of transition (from F75 milk to either F100 milk, or ready-to-use therapeutic food), provided they were clinically stable as judged by the treating physician, and had written informed consent provided by the caregiver. Exclusion criteria included those who were clinically unstable; less than 5 kg bodyweight; with neurological or oro-facial abnormalities that would explain poor feeding; where the caregiver was unwilling to learn the HIV status of the child; severe anaemia (haemoglobin <6 g/dL at enrolment); if they had an underlying condition which would put the child at undue risk of failing study completion or would interfere with analysis of study results; or if they had a contraindication to any of the trial treatments. Once children had initiated transition, caregivers were asked to provide written informed consent and participants were randomly allocated to an IMP or continuation of standard of care. Randomisation codes were pre-prepared by the trial statistician following permuted blocks to ensure balance across groups. A sealed envelope was opened for each participant at the time of recruitment to ensure allocation concealment. TAME was non-blinded for caregivers and ward staff, but laboratory scientists were blinded to intervention arm. All children received standard of care according to current WHO guidelines, which included universal use of antibiotics. Demographic information including the caregiver-reported biological sex of the child was collected at baseline. No information on gender was collected.

### Investigational medicinal products (IMPs)

All IMPs were chosen as therapies directed at restoration of the mucosal barrier and tight junction damage that occurs in malnutrition enteropathy, which is histologically similar to environmental enteropathy. The goal was to reverse the cascade of downstream inflammatory derangements seen in SAM. The intervention duration of 14 days was a pragmatic choice for this proof-of-concept trial, providing sufficient time to expect a biological effect, whilst minimising the additional time that caregiver-child pairs were kept in hospital. The IMPs chosen for this study were oral budesonide, oral colostrum, oral N-acetylglucosamine, and subcutaneous teduglutide.

1) **Budesonide** is a corticosteroid that reduces inflammation through direct local action on inflammatory cells in the gut and has extensive first-pass elimination. This reduces the systemic side-effects following oral administration. It is standard therapy for Crohn’s disease.
2) **Bovine colostrum** is a nutraceutical, which is generally regarded as safe and freely available as food supplements in health food shops. Colostrum is the first milk secreted by the cow and contains nutrients and immunoglobulins, and is higher in protein and nutritional and growth-promoting bioactive compounds than milk. It has been shown to reduce the epithelial permeability seen in heat shock in adults, and to have an effect on human health.^20^ Benefits for children with SAM may occur through the protective and anti-inflammatory properties of the immunoglobulins, which are the largest fractional protein component, as well as growth factors and hormones which encourage intestinal differentiation and proliferation. Bovine colostrum also contains large amounts of exomes and extracellular vesicles enriched with many miRNAs and proteins involved in the immune response and growth.
3) **N-acetylglucosamine** is an amide derivative of glucose and is present on every cell surface. It is a substrate for synthesising glycosaminoglycans—polysaccharides that protect the bowel mucosa from toxic damage. N-acetylglucosamine is a major component of the mucus produced by goblet cells which protects the intestinal lining, and its synthesis is impaired in patients with inflammatory bowel disease (IBD). Its administration has been shown to induce disease remission in a pilot study in paediatric IBD cases.^21^
4) **Teduglutide** is a long-acting analogue of glucagon-like peptide-2 (GLP-2), a protein secreted by the distal small intestine and colon which preserves mucosal integrity by promoting growth and repair of the intestinal epithelium. It has proven efficacy in intestinal failure associated with short bowel syndrome, including improving absorption and reducing the need for parenteral support.^22^
Full details of dosing and timing of the IMPs are shown in Supplementary Table S2.

### Laboratory methods

Luminex multiplex analysis was carried out using a 25-plex custom Luminex panel [Bio-techne Corporation (R&D), Minneapolis, MN, USA] as detailed in Supplementary Table S1 as per manufacturer’s instructions. Plasma samples were run in singlicate at 1:2 dilution using a MAGPIX reader (Luminex Corp, Austin, TX, USA).

ELISAs for stool α1-AT (Immunochrom GmbH, Germany), neopterin (Arigo Biolaboratories, Taiwan), and myeloperoxidase (Immundiagnostik, Germany) were carried out following dilution of 100 mg stool in wash buffer; following centrifugation, the ELISA was carried out on the supernatant. CRP, sCD14, sCD163 were quantified in plasma with ELISA (R&D Quantikine, Minneapolis, MN, USA), and LBP (R&D Duoset, Minneapolis, MN, USA) according to the manufacturer’s instructions.

Lipopolysaccharide (LPS, also known as endotoxin) was measured using the Limulus amoebocyte lysate (LAL) assay (Associates of Cape Cod Inc., MA, USA), which uses clotting components harvested from the blood of horseshoe crabs.^23^ Plasma was diluted 1:10 with endotoxin-free water, heated to 70 °C for 30 min, and then run according to the manufacturer’s specification with readings taken using a kinetic plate reader.

### Sample collection

Blood and stool were collected in-hospital at baseline and once during follow-up, between day 15 and 19. Blood (maximum 4 mL on any occasion and total not exceeding 2 mL/kg over two weeks) was collected in endotoxin-free EDTA tubes, then centrifuged to recover plasma, which was stored at −80 °C until further analysis. Stool was collected by study nurses or caregivers into plain tubes without fixative and placed in a cool box. Samples were sent to the laboratory within 4 h and stored at −80 °C until analysis. HIV testing was conducted at baseline.

### Statistical methods

This manuscript presents tertiary outcomes of the TAME trial. The analysis of tertiary endpoints followed the same approach as used for our primary and secondary endpoints, as detailed in our protocol^17^ and a statistical analysis plan. Day 15 biomarkers were analysed by trial arm using an ANCOVA model, adjusted for baseline values and several pre-specified covariates: HIV status (positive/negative), sex (male/female), oedema (yes/no), WHZ (continuous), diarrhoea (yes/no), and site (Zimbabwe/Zambia). Following assessment with Tukey’s ladder of powers, soluble biomarkers were normalised by log_10_ transformation. Where measurements were below the limit of quantification of the assay, a value was assigned according to the following formula^24^: assigned value = lowest level of detection/sqrt (2). Correlations between normalised biomarkers were calculated using Pearson’s correlation coefficient. Differential correlation was conducted using the Fisher’s Z transformation of the Pearson correlation coefficient in each randomiseed group, with significance determined by Fisher’s Z test.

Dimensional reduction of the biomarker data was subsequently undertaken using two principal component analyses (PCA): the first included systemic inflammatory, endothelial inflammatory/activation, and growth factor biomarkers; the second included all enteropathy biomarkers (α1-AT, neopterin, myeloperoxidase, glucagon-like peptide 2, and intestinal fatty acid-binding protein). Biomarkers with a missingness of >10% were not included in the PCA. Following normalisation as described above, biomarkers were zero-centred (i.e., each biomarker’s values were universally adjusted so that the mean was zero) and standardised (universally adjusted so that the standard deviation was 1) prior to PCA. The PCA was carried out on the day 15 (i.e., post-intervention) data, and scores for each component were generated for the day 15 and baseline results from this day 15 structure. The PCA was carried out using a complete-case approach. For the larger PCA of systemic biomarkers, components with an eigenvalue of >2 were retained. For the smaller PCA of enteropathy biomarkers, components with an eigenvalue of >1 were retained. When considering the biological interpretation of each component, a loading of >0.20 was used as a cutoff to identify the biomarkers contributing to each component. Analysis of day 15 PCA biomarkers by trial arm used an ANCOVA model, adjusted for the baseline PCA value, and the same pre-specified covariates: HIV status (positive/negative), sex (male/female), oedema (yes/no), WHZ (continuous), diarrhoea (yes/no), and site (Zimbabwe/Zambia).

Partial least squares (PLS) path modelling was used to further explore the relationship between PCA components by assessing the relationship between the gut components and the systemic components, along with a set of covariates. Any impact of the randomisation was assessed by comparing one intervention group with the standard of care arm, with the base model tested shown in Supplementary Figure S1.

As pre-specified in the protocol, the α was set at 0.1 for all analyses, consistent with an exploratory phase II trial. All results were therefore estimated with a 90% confidence interval. All these tertiary endpoints are considered exploratory, and the trial was not powered to base conclusions on these results. Consequently, results were not altered following correction for multiple outcomes. But to highlight this type 1 error risk, aiding the interpretation of results, adjusted p values derived from a Benjamini–Hochberg correction are shown. Statistical analysis was carried out using Stata v18 (StataCorp., College Station, TX), except for the differential correlation analysis, which was carried out using R Statistical Software (v4.3.1; R Core Team 2023).

### Sample size

The sample size was pre-specified as 225 (45 children in each arm), based on the primary outcome of the composite biomarker score.^17^ Given low mortality and little loss to follow-up, plus slow enrolment due to COVID-19 which closed one recruitment site, the trial steering committee and data monitoring and ethics committee reviewed the sample size in January 2021, and allowed a reduction in sample size to 125 (25 in each arm). This was based on a Cohen’s d effect size of 0.3, with 80% power and 90% confidence, and conservative correlation between baseline and follow-up estimates of 0.5, requiring 23 per group across 5 groups to analyse the primary outcome by ANCOVA. Allowing for 5% losses, the sample size of 115 was rounded up to 125 participants in total (25 per group).

### Ethics and trial registration

Written informed consent was gained from caregivers of participants. Ethical approval was obtained from the University of Zambia Biomedical Research Ethics Committee (006-09-17), the National Health Research Committee of Zambia, the Zambia Medicines Regulatory Authority (CT 082/18), the Joint Research Ethics Committee of Harare Central Hospital (JREC/66/19), the Medicines Control Authority of Zimbabwe (CT/176/2019), and the Medical Research Council of Zimbabwe (MRCZ/A/2458). The trial sponsor was Queen Mary University of London. The trial was registered at www.clinicaltrials.gov (NCT03716115) and the protocol was published.^17^

### Role of funders

Funders had no role in study design, data collection, data analyses, interpretation, or writing of this manuscript.

## Results

**Fig. 1:**
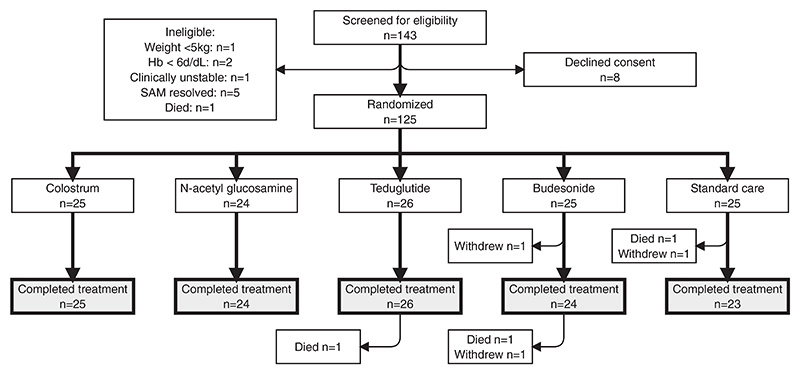
CONSORT diagram for children in the TAME trial. Children were randomised to one of five treatment arms (4 IMPs, and standard of care). Three children died, and three children withdrew. Three children exited the study prior to the day 15 endpoint, so their data were not included in the final endpoint analysis. Three children exited between day 15 and day 28, so their data were included in the current analysis.

Of 143 children who were screened for the TAME trial, 133 were eligible; 8 caregivers declined consent, leading to 125 children being enrolled and randomised after a median of 5.5 days (range 1–21) since admission to hospital with complicated SAM, as shown in Fig. 1. The baseline characteristics of enrolled children are shown in Table 1. Baseline blood and stool samples were collected from all children prior to randomisation. Randomised IMPs were provided in hospital for 14 days by study nurses. Adherence and completion were very high, with 122/124 (98%) children who survived to day 15 receiving all planned doses of IMPs, as documented by the nurses in hospital. Of 125 children with baseline samples, 2 withdrew and 1 died before collection of a follow-up blood and stool sample at day 15, therefore 122 children (98%) contributed to the post-intervention biomarker analysis.

### Biomarker results

**Fig. 2:**
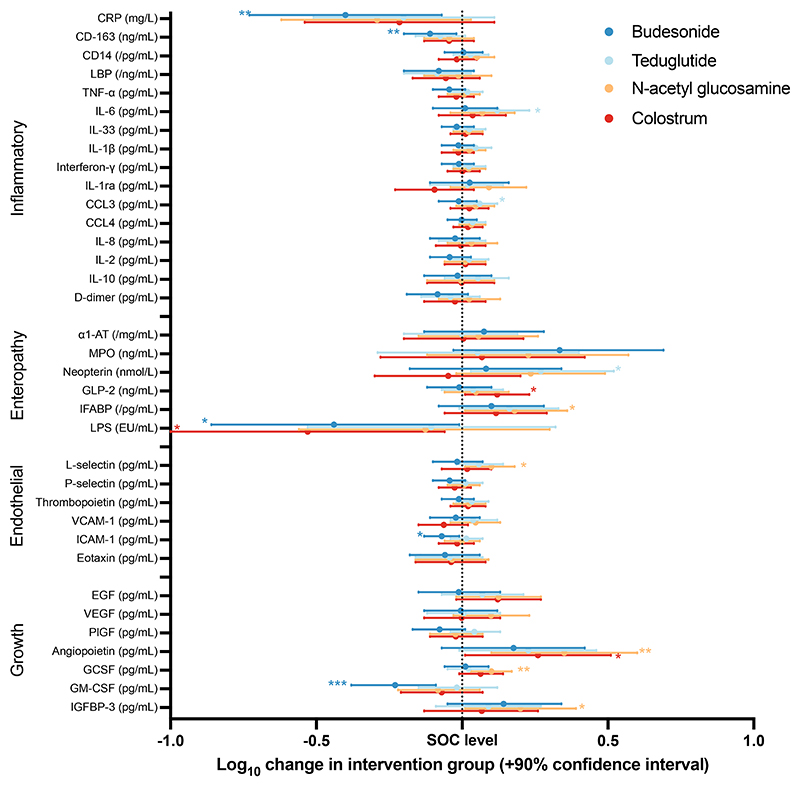
Changes of the adjusted log_10_ D15 biomarker value attributable to randomised interventions, compared to the standard of care (SOC) group. Differences are shown in the log10 concentrations of each biomarker for the budesonide, teduglutide, N-acetylglucosamine (NAG), and colostrum groups, compared to the standard of care group. Results were adjusted for sex, oedema, HIV, diarrhoea, WHZ, site, and the baseline biomarker value. A p value threshold <0.10 was pre-specified as statistically significant since this is a phase II trial and marked with *; p < 0.05 **; p < 0.01 ***. The full numerical results are shown in Supplementary Table S4.

Concentrations of biomarkers at baseline and at day 15 and the number of samples at each timepoint are summarised in Supplementary Table S3. Correlations between the individual biomarkers are shown in Supplementary Figure S2, with the differential correlations among the day 15 biomarkers by treatment group shown in Supplementary Figure S3. The results for the day 15 biomarker concentrations, adjusted for sex, oedema, HIV, diarrhoea, WHZ, site, and the baseline biomarker value, are shown in Fig. 2, with the full dataset shown in Supplementary Table S4. Children who received the corticosteroid budesonide had significantly lower concentrations of plasma CRP [−0.40 log_10_ mg/L (90% CI −0.73, −0.07)], soluble CD163 [−0.11 log_10_ ng/L (90% CI −0.20, −0.02)], LPS [−0.44 log_10_ EU/mL (90% CI −0.86, −0.01)], ICAM-1 [−0.07 log_10_ pg/mL (90% CI −0.13, −0.01)], and GM-CSF [−0.23 log_10_ pg/mL (90% CI −0.38, −0.09)] at day 15, compared with children receiving standard care. Children who received N-acetylglucosamine had higher day 15 concentrations of G-CSF [0.10 log_10_ pg/mL (90% CI 0.03, 0.17)], IGFBP-3 [0.20 log_10_ pg/mL (90% CI 0.01, 0.39)], angiopoietin [0.35 log_10_ pg/mL (90% CI 0.10, 0.60)], IFABP [0.18 log_10_ pg/mL (90% CI 0.01, 0.36)], and L-selectin [0.10 log_10_ pg/mL (90% CI 0.01, 0.18)], compared with children receiving standard care. Children who received colostrum had higher post-intervention GLP-2 [0.12 log_10_ ng/mL (90% CI 0.01, 0.23)] and angiopoietin [0.26 log_10_ pg/mL (90% CI 0.01, 0.51)], and lower LPS concentrations [−0.53 log_10_ EU/mL (90% CI −1.01, −0.06)], compared with children receiving standard care. Children who received teduglutide had higher plasma neopterin [0.27 log_10_ nmol/L (90% CI 0.03, 0.52)], IL-6 [0.12 log_10_ pg/mL (90% CI 0.02, 0.23)], and CCL3 [0.06 log_10_ pg/mL (90% CI 0.00, 0.12)] compared with children receiving standard care.

Differential correlation network analysis showed that there were several significant changes in correlations between groups, as shown visually in Supplementary Figure S3. In the teduglutide group, there were stronger relationships between IL-6 and several other biomarkers compared with the SOC group. In the NAG group D-dimer was more weakly associated with several other pro-inflammatory markers compared with the SOC group.

### Principal component analysis

**Fig. 3:**
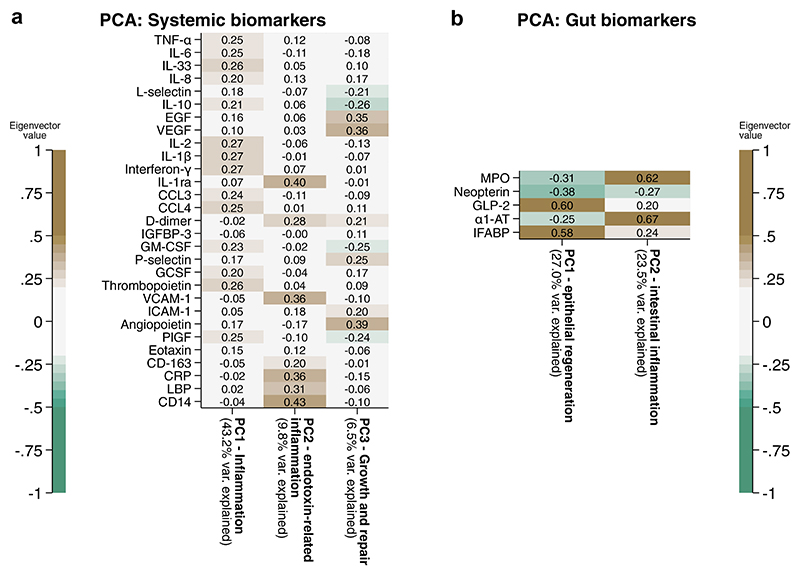
PCA analysis of a) day 15 systemic markers (N = 125) and b) day 15 enteropathy markers (N = 125). Biomarkers were log_10_ transformed, standardised, and normalised before undergoing PCA. Eigenvector values, which correspond to a coefficient of orthogonal projection attributable to each biomarker following transformation for that component, are shown.

A PCA of plasma biomarkers at day 15 was used to reduce data dimensionality. LPS was not included in the PCA analysis due to missingness >10% (Supplementary Table S2). The PCA identified three components with an eigenvalue >2, with the results shown in Fig. 3a. The full PCA plots are shown in Supplementary Figure S5. The loadings of each factor were used to biologically interpret the principal components. The first systemic biomarker component was pro-inflammatory, with top positive loadings of TNF-α, IL-6, IL-33, IL8, IL-2, IL-1β, and interferon-γ, as well as the macrophage activation proteins CCL3 and CCL4. The second systemic biomarker component was also pro-inflammatory, and captured pathways associated with host-endotoxin responses, containing positive loadings of soluble CD14, lipopolysaccharide binding protein, CRP, soluble CD163, IL-1ra, and VCAM-1. The third systemic biomarker component represented tissue restoration, containing the growth factors epidermal growth factor (EGF), vascular endothelial growth factor (VEGF), and angiopoietin, as well as d-dimer, P-selectin, and negative loading of L-selectin, IL-10, GM-CSF, and PlGF.

A PCA was similarly carried out on the gut biomarkers, resulting in two principal components with an eigenvalue of >1; their loadings are shown in Fig. 3b, and detailed in Supplementary Figure S6. Gut component 1 had strong positive loadings of plasma GLP2 and IFABP, and a negative loading of faecal myeloperoxidase, neopterin, and alpha-1-antitrypsin (α1-AT); this GLP2-dominant component likely represents increased signalling for intestinal epithelial regeneration. Gut component 2 had strong positive loadings of MPO, α1-AT and IFABP, with negative contribution from neopterin; this likely represents intestinal inflammation and damage, with the loadings of MPO and neopterin suggesting effects are mediated more by neutrophils than macrophages.

Analysis of covariance (ANCOVA) analysis was used to assess the effect of the randomised interventions on the day 15 PCA scores (Table 2), adjusting for baseline PCA score, HIV, site, oedema, WHZ, sex, and presence of diarrhoea. Budesonide and colostrum were associated with reduced values of the pro-inflammatory systemic component 2, suggesting a modulation of host endotoxin responses, and N-acetylglucosamine was associated with increased values of systemic component 3, suggesting recovery of growth factors and tissue repair. Teduglutide had no significant effect on any of the principal components.

### PLS path modelling

**Fig. 4:**
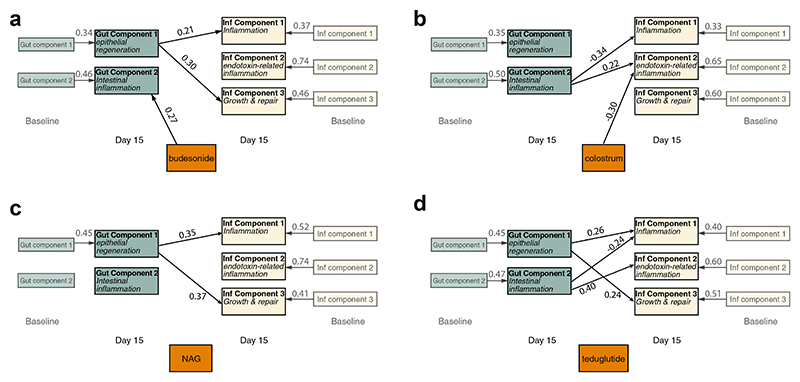
PLS path modelling of the relationship between the components and the effect of the trial intervention by a) budesonide (N = 25), b)colostrum (N = 25), c) N-acetlyglucosamine (NAG) (N = 24), and d) teduglutide (N = 26) compared with standard of care (N = 25). The relationship of the PCA components (detailed in Fig. 3) are assessed for each of the trial arms, compared with the standard of care. Results displayed are the standardised path coefficients, which are shown in full in Supplementary Table S5. Paths are displayed here is the p < 0.10 for the path coefficient. In addition to the above, HIV, site, oedema, diarrhoea, and WHZ was added and connected to each of the D15 components (covariates not shown). The full model tested is shown in Supplementary Fig. S1.

PLS path modelling was used to explore the relationships between the principal components in each of the intervention arms compared with standard of care, with results shown graphically in Fig. 4, and full breakdown shown in Supplementary Table S5. In all cases the baseline biomarker concentrations had the strongest associations with day 15 biomarker concentrations. There were significant interactions in all randomised groups between the enteropathy components and the systemic components, showing the interdependence of the gut and systemic inflammatory processes in SAM. This mirrored the correlations seen between biomarkers from different body systems in Supplementary Figure S2. The effect of the randomised group on the individual components closely mirrored the results from ANCOVA modelling in Table 2, with an additional effect of budesonide on gut component 2.

## Discussion

This study explored the impact of gut-targeted interventions on systemic and faecal biomarkers of inflammation and tissue repair, in children admitted to hospital with complicated SAM in a phase II randomised multi-centre trial in sub-Saharan Africa. All four interventions aimed at improving enteropathy appeared to show evidence of benefits beyond the gut. Budesonide led to reductions in a suite of systemic pro-inflammatory biomarkers, while colostrum and N-acetylglucosamine led to restoration of growth factors and tissue repair. Given the deleterious effects of inflammation in children with malnutrition, which drives tissue damage and prevents epithelial restoration, these interventions showed intriguing evidence of ameliorating pathological processes that hinder recovery from SAM. Together with teduglutide, which showed effects on the primary enteropathy outcome previously reported,^19^ all four interventions show laboratory evidence of distinct mechanisms of action on the interplay between inflammation and growth recovery, which could plausibly lead to clinical benefits in children with SAM.

Increased inflammation is likely to be central to the damaging multisystem processes that characterise SAM.^8^ Children with SAM demonstrate altered innate and adaptive immune responses with elevated systemic inflammatory markers.^25^ Higher inflammatory markers are associated with mortality and readmission to hospital,^9,10,26^ and lower growth velocity.^13^ Higher systemic and vascular inflammation continues for at least 48 weeks post-discharge, despite nutritional recovery, and the presence of increased growth factors in the face of this pro-inflammatory milieu is predictive of better outcomes.^9^ It is therefore becoming apparent that children with SAM could benefit from interventions which reduce inflammation and restore tissues, both during hospitalisation, and also in the year following discharge. Although several trials have examined the effect of medications or feeds aimed at improving the gut in SAM, little has changed in the management of children with SAM since WHO guidelines were introduced in 1999. The current trial aimed to specifically combat inflammation using several therapies targeting the gut.

Budesonide, an orally administered glucocorticoid with high first-pass metabolism and minimal systemic absorption, is used to limit local intestinal inflammation in inflammatory bowel disease, while limiting systemic effects. In the current study, 2 weeks of budesonide led to lower concentrations of CRP, CD163, and ICAM compared with the standard of care (SOC) arm, with a corresponding reduction in the pro-inflammatory systemic component 2 in this group. The exact mechanism for these changes in systemic markers is unclear, since oral budesonide theoretically acts locally on the intestine, and should have minimal extra-intestinal effects. In selecting oral budesonide, we hypothesised that the beneficial systemic effects are principally indirect, by improving the local inflammation that characterises malnutrition enteropathy. Therefore this would reduce the translocation of antigenic substances across the damaged gut barrier. However, there is also some evidence for systemic effects of local steroids in other diseases. For example, inhaled budesonide in COPD has a systemic effect similar to methylprednisolone,^27^ and cases of oral budesonide causing iatrogenic Addison’s disease have been reported.^28^ PLS path modelling suggested that budesonide directly affects gut component 2, which contains inflammatory biomarkers such as myeloperoxidase and alpha-1 antitrypsin (Fig. 4). In the PCA, budesonide had no effect on the Gut components, but led to a reduction in one of the systemic components, which reflected inflammatory pathways associated with host-endotoxin responses (Table 2). It is therefore plausible that improvements in circulating pro-inflammatory biomarkers occur through a more direct systemic effect, despite the high first-pass metabolism of budesonide.

N-acetylglucosamine (NAG), a naturally occurring amino sugar which can be used as a precursor for epithelial glycosaminoglycan synthesis, was associated with an increase in the growth factor-containing principal component by day 15. As a sugar which is depleted in patients with IBD,^29^ NAG can improve IBD symptoms in children^21^ and inhibits inflammation and neurogeneration in adults with multiple sclerosis,^30^ through controlling the N-linked glycans in cells which subsequently help to suppress T-helper-1 cells. This nutraceutical product, along with budesonide, is one of the cheapest and easiest to administer, as the product can be kept at room temperature and is given orally. Bovine colostrum, the foremilk rich in immunoglobulins, growth factors and other bioactive components with anti-inflammatory properties, decreased values of the pro-inflammatory systemic component 2 compared to the SOC arm. In addition, colostrum led to higher levels of circulating GLP2 and angiopoietin, which are markers of increased epithelial turnover and repair.^31^ Colostrum contains extracellular vesicles which have been shown to induce intestinal repair and improve intestinal permeability in mice. It should be noted that among the extensive nutritional components of bovine colostrum, it is a source of GLP2, although its bioavailability is poor. Colostrum also led to significant reductions in circulating LPS, suggesting that improved epithelial repair may reduce translocation of LPS, although formal gut permeability testing, such as urinary excretion of lactulose:rhamnose, would be required to confirm this hypothesis.

The PLS path modelling examining the relationship between the enteropathy and inflammation components for each trial arm showed that, despite the intervention being aimed at reducing enteropathy, changes occurred in both gut and systemic inflammatory markers, showing the interdependence between gut and systemic markers. This highlights the central role that malnutrition enteropathy may play in driving the inflammatory changes seen in SAM,^8^ and that utilising interventions targeting the gut may have downstream systemic effects. Few other trials have evaluated interventions targeting enteropathy in complicated SAM, although smaller trials of probiotics in Malawi,^32^ microbiota-directed foods in Bangladesh,^33^ pancreatic enzymes in Malawi,^34^ and a proof-of-concept trial of mesalazine in Kenya^35^ have all shown benefits. Despite these promising initial studies, interventions have not yet progressed further to Phase III trials to assess effectiveness, and despite their recent 2023 update, the WHO guidance on the treatment of complicated SAM has not substantially changed in the last few decades, although it does now recognise the research need to ‘study non-antibiotic pharmacologic and other medical interventions’.

Overall, most biomarkers significantly changed over a 2-week gut-targeted intervention: All markers of systemic inflammation and innate immune activation, except D-dimer, decreased. Markers of vascular inflammation and activation also declined (Supplementary Table S2). Collectively, this is likely to represent a partial natural resolution of the inflammatory processes acutely present in children with SAM as children recover, and may reflect the fact that many of these children present with infections. That some inflammatory biomarkers are even lower and some growth factors are higher in the treatment arms, compared to standard care, highlights the additional benefits arising from several of the interventions. Children with complicated SAM remain vulnerable and in need of potential therapeutic intervention long after their inpatient rehabilitation: a previous study showed that inflammatory markers in children with SAM remain higher than adequately-nourished community controls over the following year with current standard care. Additionally, poorer outcomes over the following year were associated with higher PCA component scores representing systemic and vascular inflammation and lower component scores representing growth factors at baseline; increases in GLP-2 and I-FABP were both associated with improved outcome and each log_10_ rise of VEGF was independently associated with a 50% reduction in mortality or readmission.^9^ A suggestion of this same interplay between inflammatory markers and growth factors was seen in the current study, in the PCA analysis by treatment group as well as negative correlations seen in simple correlation analyses (Supplementary Figure S2). Both colostrum and budesonide reduced the systemic component 2, primarily composed of pro-inflammatory biomarkers. In the case of colostrum, the reduction of LPS suggests the mechanism involved may be a reduction of intestinal translocation of bacterial products and subsequent immune activation, and the effects of budesonide are likely mediated by its anti-inflammatory properties. N-acetylglucosamine increased the level of systemic component 3, primarily composed of growth factors, and individually increased IGFBP-3, angiopoietin, and G-CSF, consistent with increased epithelial glycosaminoglycan synthesis, and likely reflecting increased repair and regeneration. This interplay between inflammation and growth/tissue accretion may well represent a therapeutic target in itself, suggesting that interventions that push the balance towards repair and growth could potentially be beneficial, given the respective concentrations of these biomarkers are associated with outcomes. The trial tested a two-week intervention period during hospitalisation. Given the evidence that abnormalities in inflammatory markers remain for at least a year post-discharge,^9^ children might well benefit from prolonged therapy but further studies will be needed to assess any longer-term impact on biomarkers and clinical outcomes.

There are limitations to this analysis. This was a relatively small trial and in these exploratory analyses, which are tertiary trial outcomes. We did not base our inferences on p-values or on single biomarkers, and looked instead at the consistency of effects and based most findings on our higher dimensional analyses. With these exploratory analyses, particularly with conclusions on single biomarkers, there is a risk of type 1 error. We have included a corrected p-value to highlight this and to aid in the interpretation of results (Supplementary Table S4). We have consequently tried not to base inferences on single biomarkers, and larger adequately powered trial will be needed before any definitive conclusions regarding individual biomarkers could be reached. Additionally, there are some limitations in the applicability of the data to real-world situations, since adherence to these drugs was high in a clinical trial setting. Furthermore, as children were recruited following stabilisation, they are not reflective of all children who present with SAM, as emphasised by the low 28-day mortality rate of under 3%, compared with the higher mortality rates reported elsewhere.^3^ Data on other factors, such as home water, sanitation, and hygiene facilities, which have the potential to impact the severity of enteropathy, was not collected, but we would expect such factors to be broadly balanced across randomisation groups. It is possible that sub-groups, such as children with oedema or diarrhoea, responded differently to the interventions, but this trial was not powered to explore these questions.

In conclusion, we show that there is biological plausibility in extending the range of therapeutic interventions in the very high-risk population of children with complicated SAM. In this phase II randomised controlled trial, interventions designed to heal the gut led to reductions in systemic inflammation and increase in growth factors and biomarkers of tissue repair. Given that the interplay between systemic inflammation and tissue accretion is central to recovery from SAM,^9^ the next step is to test these strategies in a larger phase III trial to evaluate whether laboratory improvements are accompanied by lower mortality and readmission to hospital, and better growth recovery.

## Supplementary Material

**Appendix A. Supplementary data**

Supplementary data related to this article can be found at https://doi.org/10.1016/j.ebiom.2024.105478.

Supplementary Tables S1–S5 and Figures S1–S6

Trial protocol (v3.0, 16th December 2020)

## Figures and Tables

**Table 1:** Baseline characteristics of children in the TAME trial by randomisation group.

	Randomisation										
	Colostrum		NAG		Teduglutide		Budesonide		Standard care		Total
	N = 25		N = 24		N = 26		N = 25		N = 25		N = 125
Age (months)	20 (15, 23)		19 (14, 23)		18 (12, 20)		17 (13, 22)		16 (13, 27)		18 (13, 22)
Male	13 (52%)		16 (67%)		16 (62%)		11 (44%)		15 (60%)		71 (57%)
Site											
Zambia	12 (48%)		12 (50%)		13 (50%)		12 (48%)		13 (52%)		62 (50%)
Zimbabwe	13 (52%)		12 (50%)		13 (50%)		13 (52%)		12 (48%)		63 (50%)
HIV positive	4 (16%)		4 (17%)		6 (23%)		2 (8%)		8 (32%)		24 (19%)
Days to recruitment	6 (5, 8)		5 (3, 6)		5 (4, 7)		4 (3, 5)		5 (3, 7)		5 (3, 7)
MUAC (cm)	11.4 (10.8, 11.9)		11.5 (11.3, 12.9)		11.6 (11.0, 12.3)		12.0 (11.4, 12.6)		11.7 (11.0, 12.3)		11.6 (11.0, 12.5)
WAZ	−3.65 (−5.45, −3.25)		−4.05 (−4.71, −2.86)		−3.39 (−4.32, −2.75)		−2.99 (−3.86, −2.36)		−3.39 (−4.50, −2.70)		−3.59 (−4.50, −2.74)
WHZ	−2.72 (−4.19, −2.35)		−2.59 (−3.74, −1.24)		−2.22 (−3.34, −1.44)		−2.21 (−2.69, −1.59)		−2.62 (−3.60, −1.89)		−2.38 (−3.47, −1.59)
*WHZ (oedematous)*	−*2.57* (−*3.47*, −*2.15*)		−*2.26* (−*3.34*, −*1.22*)		−*1.78* (−*2.35*, −*1.37*)		−*2.01* (−*2.34*, −*1.58*)		−*2.10* (−*3.44*, −*1.33*)		−*2.21* (−*3.15*, −*1.51*)
HAZ	−3.07 (−3.82, −2.72)		−4.11 (−4.63, −2.73)		−3.29 (−4.38, −2.66)		−3.04 (−3.44, −2.21)		−3.17 (−4.41, −1.98)		−3.19 (−4.39, −2.39)
Oedema											
None	8 (32%)		7 (29%)		5 (19%)		7 (28%)		5 (20%)		32 (26%)
+	7 (28%)		6 (25%)		15 (58%)		8 (32%)		10 (40%)		46 (37%)
++	10 (40%)		10 (42%)		5 (19%)		10 (40%)		9 (36%)		44 (35%)
+++	0 (0%)		1 (4%)		1 (4%)		0 (0%)		1 (4%)		3 (2%)
Birthweight (kg)	3.00 (2.70, 3.20)		2.80 (2.58, 3.05)		2.92 (2.70, 3.44)		3.20 (2.80, 3.40)		3.10 (2.60, 3.40)		3.00 (2.70, 3.40)
Premature (<37 weeks)	6 (24%)		4 (17%)		6 (24%)		4 (16%)		2 (8%)		22 (18%)
Acute diarrhoea	1 (4%)		0 (0%)		1 (4%)		0 (0%)		1 (4%)		3 (2%)
Persistent diarrhoea	0 (0%)		0 (0%)		1 (4%)		1 (4%)		1 (4%)		3 (2%)
Pneumonia	0 (0%)		1 (4%)		1 (4%)		3 (12%)		3 (12%)		8 (6%)
TB	5 (20%)		3 (13%)		6 (23%)		4 (16%)		6 (24%)		24 (19%)
UTI	0 (0%)		0 (0%)		1 (4%)		0 (0%)		0 (0%)		1 (1%)
Cerebral palsy	0 (0%)		2 (8%)		2 (8%)		2 (8%)		0 (0%)		6 (5%)

This table shows the baseline (i.e., at randomisation) demographics of children with SAM (N = 125) by randomised group. Continuous data are displayed as the median value, with lower and upper interquartile range. Categorical data show the number (N) in that category, and the percentage of the total randomisation group. Persistent diarrhoea is defined as diarrhoea present for ≥14 days. The WHZ (oedematous) in italics is a subgroup that shows the results of just the children with oedematous malnutrition. NAG: N-acetylglucosamine; MUAC: mid-upper arm circumference; WHZ: weight-for-height z-score; HAZ: height-for-age z-score; TB: tuberculosis; UTI: urinary tract infection.

**Table 2:** Differences in component scores derived from biomarkers measured at day 15 between treatment group and the standard of care (SOC).

	Colostrum			NAG			Teduglutide			Budesonide	
	Change versus SOC	90% CI		Change versus SOC	90% CI		Change versus SOC	90% CI		Change versus SOC	90% CI
**Systemic component** **1** – *inflammation*	0.19	(−0.93, 1.31)		0.61	(−0.50, 1.71)		0.65	(−0.43, 1.72)		−0.42	(−1.52, 0.69)
**Systemic component** **2** – *endotoxin-related*	−0.70*	(−1.35, −0.05)		−0.03	(−0.67, 0.62)		−0.29	(−0.91, 0.34)		−0.72*	(−1.38, −0.07)
**Systemic component** **3** – *growth and repair*	0.39	(−0.23, 1.00)		0.73*	(0.12, 1.34)		0.25	(−0.34, 0.83)		0.20	(−0.41, 0.81)
**Gut component** **1** – *epithelial regeneration*	0.36	(−0.18, 0.89)		0.02	(−0.50, 0.54)		0.04	(−0.48, 0.56)		−0.16	(−0.70, 0.38)
**Gut component** **2** – *intestinal inflammation*	0.30	(−0.20, 0.81)		0.40	(−0.10, 0.90)		0.05	(−0.45, 0.55)		0.45	(−0.07, 0.97)

Results obtained using ANCOVA with adjustment for baseline PCA score, HIV, site, oedema, WHZ, sex, and presence of diarrhoea. Pre-specified significance threshold, p value < 0.10 equivalent to 90% confidence intervals (CI). ***p-value < 0.01, **p-value < 0.05,

* p-value < 0.10.

## Data Availability

The TAME dataset with demographics and primary/secondary outcomes is available here: https://doi.org/10.6084/m9.figshare.24442699. The dataset for tertiary outcomes can be provided by the authors pending scientific review and a completed material transfer agreement. Requests for this dataset should be submitted to the corresponding author.

## Research in context

### Evidence before this study

Children admitted with severe acute malnutrition (SAM) mostly reside in lower- and middle-income countries. There is a high mortality attributed to this condition, and despite this, the underlying pathophysiology is poorly understood. Few novel treatments have been trialled in this patient population, and plasma biomarkers have rarely been interrogated with multiplexed biomarker analyses. Children with SAM have higher plasma biomarkers of systemic and vascular inflammation compared with community control children without acute malnutrition, and elevated inflammation persists for at least 48 weeks after discharge. The higher concentrations of several biomarkers is associated with poorer outcomes. The TAME trial has shown that four new interventions aimed at treating the gut dysfunction seen in children with severe malnutrition, were well tolerated, and one intervention (teduglutide) positively impacted a composite biomarker score representing intestinal inflammation.

### Added value of this study

In this analysis of tertiary outcomes of the randomised controlled TAME trial, we show that two interventions aimed at treating gut dysfunction, budesonide and colostrum, improved systemic and vascular inflammation biomarkers. Administration of N-acetylglucosamine, a precursor for epithelial glycosaminoglycan synthesis, increased biomarkers of epithelial regeneration and circulating growth factors. This potentially represents improved repair and tissue accretion, and concentrations of some of these biomarkers have previously been associated with improved linear growth and outcomes following discharge. Finally, with structural modelling, we analysed the interplay between systemic inflammation and markers of growth and epithelial regeneration, which suggested that higher levels of inflammation can prevent the growth and regenerative processes in these children.

### Implications of all the available evidence

Our results suggest that interventions could reduce systemic and vascular inflammation in children with SAM, which in other studies exists up to at least 48 weeks post-discharge. It is also possible to increase the levels of some growth factors, which have previously been shown to be associated with better outcomes. Growth factors were negatively related to the concentrations of systemic/vascular inflammation markers, highlighting the apparent relationship between the two. These data now need to be taken forward in further trials to see if a longer intervention period leads to more persistent changes in biomarkers, whether combined interventions may be better than single interventions, and whether changes in biomarkers translate into better clinical outcomes in these high-risk children.
