# Supplementary material for: Therapeutic interventions targeting enteropathy in severe acute malnutrition modulate systemic and vascular inflammation and epithelial regeneration
Source: EBioMedicine. Author manuscript; Available in PMC 2025 Jan 8. (PMC11697704; doi:10.1016/j.ebiom.2024.105478)
Supplement: Supplementary Tables S1–S5 and Figures S1–S6 [file EMS202076-supplement-Supplementary_Tables_S1_S5_and_Figures_S1_S6.pdf]

Supplementary Material

Tables

Supplementary Table 1: List of biomarkers analysed....2
Supplementary Table 2: Investigational medicinal products and their preparations....3
Supplementary Table 3: Biomarkers analysed, number of samples, and results shown at baseline, day 15 (D15) and the overall results....4
Supplementary Table 4: Changes of the D15 biomarker concentration attributable to randomized intervention, over the standard of care (SOC) group....5
Supplementary Table 5: Standardised path coefficients from PLS-Path modelling by each randomisation group....6

Figures

Supplementary Figure 1: Base model tested in structural path modelling....7
Supplementary Figure 2: Correlation coefficients between the biomarker levels at Day 15....8
Supplementary Figure 3: Differential correlation network analysis between groups....9
Supplementary Figure 4: Changes of the adjusted log10 D15 biomarker value attributable to randomized intervention, over the standard of care (SOC) group....10
Supplementary Figure 5: Principal component analysis plots of the all-timepoint results showing the individual participants split by randomization....11
Supplementary Figure 6: PCA plots for the gut components at all timepoints....12

	Protein ID	Full name	Method	Sample type	Limit of detection (LOD)
Inflammatory	**CRP**	C-reactive protein	ELISA	Plasma	0.004 mg/L
	**sCD14**	Soluble CD14	ELISA	Plasma	900000 pg/mL
	**LBP**	Lipopolysaccharide-binding-protein	ELISA	Plasma	200 ng/mL
	**sCD163**	Soluble CD163	ELISA	Plasma	250 pg/mL
	**TNFα**	Tumour necrosis factor-alpha	Luminex panel	Plasma	20 pg/mL
	**IL-6**	Interleukin-6	Luminex panel	Plasma	0.13pg/mL
	**IL-33**	Interleukin-33	Luminex panel	Plasma	47pg/mL
	**IL-8**	Interleukin-8	Luminex panel	Plasma	8.6pg/mL
	**IL-10**	Interleukin-10	Luminex panel	Plasma	0.72 pg/mL
	**IL-2**	Interleukin-2	Luminex panel	Plasma	13pg/mL
	**IL-1β**	Interleukin-1β	Luminex panel	Plasma	15pg/mL
	**IFNγ**	Interferon-gamma	Luminex panel	Plasma	71pg/mL
	**IL-1ra**	Interleukin -1 receptor antagonist	Luminex panel	Plasma	310 pg/mL
	**CCL3**	Chemokine (C-C motif) ligand 3	Luminex panel	Plasma	88pg/mL
	**CCL4**	Chemokine (C-C motif) ligand 4	Luminex panel	Plasma	650pg/mL
	**D-dimer**	D-dimer	Luminex panel	Plasma	235,000pg/mL
Endothelial	**P-selectin**	P-selectin	Luminex panel	Plasma	11900 pg/mL
	**L-selectin**	L-selectin	Luminex panel	Plasma	76000 pg/mL
	**VCAM-1**	Vascular cell adhesion molecule 1	Luminex panel	Plasma	210000 pg/mL
	**ICAM-1**	Intercellular adhesion molecule 1	Luminex panel	Plasma	3500 pg/mL
	**TPO**	Thrombopoietin	Luminex panel	Plasma	1600 pg/mL
	**Eotaxin**	Eotaxin-1 (CCL11)	Luminex panel	Plasma	29.5pg/mL
Growth	**GM-CSF**	Granulocyte-macrophage colony-stimulating factor	Luminex panel	Plasma	2.12pg/mL
	**GCSF**	Granulocyte colony-stimulating factor	Luminex panel	Plasma	55pg/mL
	**EGF**	Epidermal growth factor	Luminex panel	Plasma	2.98 pg/mL
	**VEGF**	Vascular endothelial growth factor	Luminex panel	Plasma	23 pg/mL
	**Angiopoietin**	Angiopoietin-1	Luminex panel	Plasma	30 pg/mL
	**PlGF**	Placental growth factor	Luminex panel	Plasma	1pg/mL
	**IGFBP-3**	IGF-binding protein 3	Luminex panel	Plasma	640 pg/mL
Enteropathy	**GLP-2**	Glucagon-like peptide 2	ELISA	Plasma	0.3 ng/mL
	**IFABP**	Intestinal fatty-acid binding protein	ELISA	Plasma	153 pg/mL
	**MPO**	Myeloperoxidase	ELISA	Stool	3.99 ng/mL
	**Neopterin**	Neopterin	ELISA	Stool	4.7 nmol/L
	**α1-AT**	Alpha-1-antitrypsin	ELISA	Stool	0.007 mg/mL
	**LPS**	Lipopolysaccharide (also called endotoxin)	LAL assay	Plasma	0.002EU/mL

Supplementary Table 1: List of biomarkers analysed

The Luminex panel was one single 25-plex panel (Assay code: UgRYKY2M; R&D Systems, Minneapolis, USA). ELISAs were run separately.

ELISA kits (R&D Systems, Minneapolis, USA) were used to analyse CRP (cat number:SCRP00) and sCD14 (cat number: DC140) at 1:200 dilution; and CD163 (cat number: DC1630) and LBP (cat number: DY870) at 1:40 dilution. GLP-2 (Merck, Darmstadt, Germany; cat number: EZGLP2) was analysed without dilution. IFABP (HycultBiotech, Uden , The Netherlands; Cat number: HK406) was analysed following 1:2 dilution. MPO (Immunodiagnostik, Bensheim, Germany; cat number: K6630) was analysed following 1:500 dilution. α1-AT (ImmunoChrom, Heppenheim, Germany; Cat number: IC6200) was analysed following 1:12500 dilution. Neopterin (Arigobio, Hsinchu, Taiwan; Cat number: ARG80878) was analysed following 1:50 dilution.

	Colostrum	N-acetyl glucosamine	Teduglutide	Budesonide	Standard care
**Preparation**	Powder	Powder	Ampoule	Liquid	-
**Route**	Oral	Oral	Subcutaneous	Oral	-
**N**	25	25	25	25	25
**Dose:**					-
**Days 1-7**	1.5g tds	300mg tds	0.05mg/kg daily	1mg tds	
**Days 8-11**	1.5g tds	300mg tds	0.05mg/kg daily	1mg bd	
**Days 12 – 14**	1.5g tds	600mg tds	0.05mg/kg daily	0.5 mg bd	
**Cost**	£28 for 200g[1]	USD23.20 for 250g[2].	£7,307 for 28 1.25mg vials[3]	£41.19 for 20 1mg vials[3]	

Supplementary Table 2: Investigational medicinal products and their preparations

[1]Costs taken from Colostrum UK; www.neovite.com. [2]Costs taken from Blackburn Distributions; www.blackburndistributions.com, [3]Costs are taken from https://bnf.nice.org.uk and are the UK nationally agreed prices, correct as of September 2022. Tds: four times a day; bd: twice a day.

		Baseline [n=125]					Day 15 [n=122]				
				Included		Miss-ing			Included		Miss-ing
	Biomarker (pg/mL, unless stated)	Median	[IQR]	total	*of which, OOR*		Median	[IQR]	total	*of which, OOR*	
Inflammatory	CRP (mg/L)	1.823	[0.698-6.616]	n=123		2	0.908	[0.302-3.819]	n=122		0
	CD-163 (ng/mL)	1,191	[825-1,808]	n=123		2	1,098	[806-1,562]	n=122		0
	sCD14 (ug/mL)	2.2	[1.8-2.6]	n=123		2	2.0	[1.7-2.5]	n=122		0
	LBP (ng/mL)	5,982	[4,302-8,592]	n=123		2	5,815	[4,208-8,092]	n=122		0
	TNF-α	50.8	[38.5-65.1]	n=123		2	45.4	[34.5-61.7]	n=121		1
	IL-6	32.2	[11.9-40.9]	n=123		2	26.6	[7.7-35.9]	n=121	*1*	1
	IL-33	115.5	[93.9-135.1]	n=123		2	101.1	[87.1-129.7]	n=121		1
	IL-1β	54.1	[38.2-64.4]	n=123		2	47.0	[31.9-59.3]	n=121		1
	Interferon-γ	173	[143-211]	n=123		2	159	[125-206]	n=121		1
	IL-1ra	1,344	[864-2,232]	n=123		2	863	[659-1,332]	n=121		1
	CCL3	452	[241-525]	n=123		2	436	[224-497]	n=121		1
	CCL4	1,073	[920-1,222]	n=123		2	984	[861-1,149]	n=121		1
	IL-8	43.2	[33.3-59.3]	n=123		2	34.0	[26.7-45.9]	n=121		1
	IL-2	81.5	[43.8-98.9]	n=123		2	72.5	[34.4-94.0]	n=121		1
	IL-10	13.4	[8.9-17.2]	n=122		3	11.9	[6.4-15.9]	n=121		1
	D-dimer (ug/mL)	1.8	[1.1-2.4]	n=123		2	1.8	[1.3-2.7]	n=121		1
Enteropathy	α1-AT (/mg/mL)	0.114	[0.051-0.379]	n=121		4	0.341	[0.164-0.745]	n=118	*7*	4
	MPO (ng/mL)	1,361	[441-5,217]	n=121	*2*	4	838	[355-1,670]	n=118	*5*	4
	Neopterin (nmol/L)	372.4	[195.5-731.7]	n=120	*1*	5	575.6	[326.7-991.5]	n=119	*3*	3
	GLP-2 (ng/mL)	5.7	[3.7-8.0]	n=123	*1*	2	4.4	[3.0-5.9]	n=122	*1*	0
	IFABP	1,555	[795-2,680]	n=123		2	1,639	[1,027-3,152]	n=122		0
	LPS (EU/mL)	0.005	[0.002-0.015]	n=108	*47*	17	0.009	[0.004-0.034]	n=105	*34*	17
Endothelial	L-selectin (ng/mL)	777.1	[479.0-1072.9]	n=123		2	575.3	[389.2-909.9]	n=121		1
	P-selectin (ng/mL)	34	[27-40]	n=123		2	29	[24-34]	n=121		1
	Thrombopoietin	3,645	[2,976-4,175]	n=123		2	3,174	[2,680-4,114]	n=121		1
	VCAM-1 (ug/mL)	1.6	[1.2-2.1]	n=123		2	1.3	[1.0-1.8]	n=121		1
	ICAM-1	587	[370-897]	n=123		2	558	[359-777]	n=121		1
	Eotaxin	142.8	[104.6-182.1]	n=123	*5*	2	129.5	[88.4-165.7]	n=121	*5*	1
Growth	EGF	22.3	[16.8-33.4]	n=123		2	22.6	[17.4-33.4]	n=121		1
	VEGF	75.5	[57.6-104.8]	n=123		2	83.0	[63.8-118.8]	n=121		1
	PlGF	71.1	[8.1-88.2]	n=123		2	62.1	[7.2-78.6]	n=121		1
	Angiopoietin	5,583	[2,159-10,697]	n=123		2	4,433	[1,867-12,553]	n=121		1
	GCSF	197.8	[149.0-235.3]	n=123		2	184.0	[151.5-227.7]	n=121		1
	GM-CSF	24.9	[6.0-40.0]	n=123	*24*	2	20.4	[2.0-33.4]	n=121	*34*	1
	IGFBP-3 (ng/mL)	135	[68-200]	n=123		2	198	[92-341]	n=121		1

Supplementary Table 3: Biomarkers analysed, number of samples, and results shown at baseline, day 15 (D15) and the overall results

The total samples analysed is shown, and the number of this total which includes samples out of range (OOR), which were included at (limit of detection /√2) are shown. The number of samples missing, that being the number of children in the trial at that time without any result, is shown. Missingness was due to insufficiency of samples.

	Biomarker (log_{10}) in pg/mL unless stated	Colostrum				NAG				Teduglutide				Budesonide			
		D15 level compared with SOC[1]	90% CI	p-value	*Adj p-value*[2]	D15 level compared with SOC[1]	90% CI	p-value	*Adj p-value*[2]	D15 level compared with SOC[1]	90% CI	p-value	*Adj p-value*[2]	D15 level compared with SOC[1]	90% CI	p-value	*Adj p-value*[2]
Inflammatory	CRP (mg/L)	-0.21	(-0.54, 0.11)	0.28	0.91	-0.29	(-0.62, 0.03)	0.14	0.66	-0.20	(-0.51, 0.11)	0.29	0.80	-0.40**	(-0.73,-0.07)	0.046	0.54
	CD-163 (ng/mL)	-0.04	(-0.13, 0.04)	0.40	0.91	-0.04	(-0.13, 0.04)	0.41	0.76	-0.08	(-0.16, 0.01)	0.15	0.80	-0.11**	(-0.20,-0.02)	0.037	0.54
	sCD14	-0.02	(-0.08, 0.05)	0.64	0.91	0.05	(-0.01, 0.11)	0.19	0.67	0.03	(-0.03, 0.09)	0.43	0.80	0.00	(-0.06, 0.07)	0.91	0.96
	LBP (ng/mL)	-0.06	(-0.17, 0.06)	0.44	0.91	-0.01	(-0.13, 0.10)	0.85	0.90	-0.09	(-0.20, 0.03)	0.21	0.80	-0.08	(-0.20, 0.04)	0.27	0.73
	TNF-α	-0.02	(-0.08, 0.04)	0.55	0.91	0.01	(-0.05, 0.06)	0.84	0.90	0.02	(-0.04, 0.07)	0.58	0.81	-0.04	(-0.10, 0.01)	0.19	0.70
	IL-6	0.04	(-0.08, 0.15)	0.61	0.91	0.07	(-0.04, 0.18)	0.31	0.72	0.12*	(0.02, 0.23)	0.06	0.80	0.01	(-0.10, 0.12)	0.89	0.96
	IL-33	0.01	(-0.04, 0.07)	0.72	0.91	0.02	(-0.03, 0.07)	0.52	0.79	0.03	(-0.03, 0.08)	0.42	0.80	-0.02	(-0.07, 0.04)	0.56	0.96
	IL-1β	-0.01	(-0.07, 0.04)	0.70	0.91	0.03	(-0.03, 0.08)	0.45	0.76	0.05	(-0.01, 0.10)	0.16	0.80	-0.01	(-0.07, 0.04)	0.71	0.96
	Interferon-γ	0.00	(-0.05, 0.06)	0.97	0.98	0.02	(-0.03, 0.08)	0.48	0.76	0.02	(-0.03, 0.08)	0.47	0.80	-0.01	(-0.07, 0.04)	0.74	0.96
	IL-1ra	-0.09	(-0.23, 0.04)	0.24	0.91	0.09	(-0.04, 0.22)	0.25	0.67	0.02	(-0.11, 0.14)	0.81	0.90	0.03	(-0.11, 0.16)	0.75	0.96
	CCL3	0.02	(-0.04, 0.09)	0.53	0.91	0.05	(-0.02, 0.11)	0.22	0.67	0.06*	(0.00, 0.12)	0.09	0.80	-0.01	(-0.08, 0.05)	0.77	0.96
	CCL4	0.02	(-0.03, 0.07)	0.48	0.91	0.03	(-0.01, 0.08)	0.25	0.67	0.04	(-0.01, 0.08)	0.19	0.80	-0.00	(-0.05, 0.05)	0.96	0.96
	IL-8	-0.00	(-0.09, 0.08)	0.94	0.98	0.03	(-0.05, 0.12)	0.54	0.79	0.00	(-0.08, 0.08)	0.95	0.98	-0.02	(-0.11, 0.06)	0.64	0.96
	IL-2	0.01	(-0.06, 0.08)	0.79	0.92	0.01	(-0.06, 0.08)	0.81	0.90	0.02	(-0.04, 0.09)	0.55	0.80	-0.04	(-0.11, 0.03)	0.30	0.75
	IL-10	-0.00	(-0.12, 0.11)	0.98	0.98	-0.00	(-0.12, 0.11)	0.95	0.98	0.05	(-0.06, 0.16)	0.42	0.80	-0.02	(-0.13, 0.10)	0.82	0.96
	D-dimer	-0.02	(-0.13, 0.08)	0.69	0.91	0.02	(-0.08, 0.13)	0.69	0.83	-0.04	(-0.14, 0.06)	0.50	0.80	-0.08	(-0.19, 0.02)	0.17	0.70
Enteropathy	α1-AT (mg/mL)	0.00	(-0.20, 0.21)	0.98	0.98	0.06	(-0.15, 0.26)	0.65	0.83	-0.00	(-0.20, 0.19)	0.99	0.99	0.08	(-0.13, 0.28)	0.55	0.96
	MPO (ng/mL)	0.07	(-0.28, 0.42)	0.75	0.91	0.23	(-0.12, 0.57)	0.28	0.70	0.05	(-0.29, 0.40)	0.80	0.90	0.33	(-0.03, 0.69)	0.13	0.70
	Neopterin (nmol/L)	-0.05	(-0.30, 0.20)	0.75	0.91	0.24	(-0.02, 0.49)	0.12	0.66	0.27*	(0.03, 0.52)	0.07	0.80	0.08	(-0.18, 0.34)	0.60	0.96
	GLP-2 (ng/mL)	0.12*	(0.01, 0.23)	0.07	0.91	0.05	(-0.06, 0.16)	0.48	0.76	0.04	(-0.07, 0.14)	0.55	0.80	-0.01	(-0.12, 0.10)	0.89	0.96
	IFABP (/pg/mL)	0.12	(-0.06, 0.29)	0.28	0.91	0.18*	(0.01, 0.36)	0.08	0.63	0.16	(-0.01, 0.33)	0.11	0.80	0.10	(-0.08, 0.28)	0.35	0.82
	LPS (EU/mL)	-0.53*	(-1.01,-0.06)	0.07	0.91	-0.13	(-0.56, 0.30)	0.63	0.83	-0.11	(-0.53, 0.32)	0.68	0.84	-0.44*	(-0.86,-0.01)	0.09	0.63
Endothelial	L-selectin	0.02	(-0.07, 0.10)	0.74	0.91	0.10*	(0.01, 0.18)	0.06	0.63	0.06	(-0.02, 0.14)	0.25	0.80	-0.02	(-0.10, 0.07)	0.74	0.96
	P-selectin	-0.03	(-0.08, 0.03)	0.44	0.91	0.01	(-0.05, 0.06)	0.84	0.90	0.01	(-0.04, 0.07)	0.70	0.84	-0.04	(-0.10, 0.01)	0.20	0.70
	Thrombopoietin	0.02	(-0.04, 0.08)	0.55	0.91	0.02	(-0.03, 0.08)	0.46	0.76	0.03	(-0.02, 0.09)	0.31	0.80	-0.01	(-0.07, 0.04)	0.75	0.96
	VCAM-1	-0.06	(-0.15, 0.02)	0.23	0.91	0.05	(-0.04, 0.13)	0.37	0.76	0.04	(-0.04, 0.12)	0.44	0.80	-0.02	(-0.11, 0.06)	0.68	0.96
	ICAM-1	-0.02	(-0.08, 0.04)	0.65	0.91	-0.00	(-0.06, 0.06)	1.00	1.00	0.01	(-0.04, 0.07)	0.68	0.84	-0.07*	(-0.13,-0.01)	0.08	0.63
	Eotaxin	-0.04	(-0.16, 0.08)	0.61	0.91	-0.03	(-0.16, 0.09)	0.63	0.83	-0.04	(-0.16, 0.07)	0.54	0.80	-0.06	(-0.18, 0.06)	0.42	0.92
Growth	EGF	0.12	(-0.02, 0.27)	0.16	0.91	0.13	(-0.02, 0.27)	0.15	0.66	0.07	(-0.07, 0.21)	0.40	0.80	-0.01	(-0.15, 0.13)	0.89	0.96
	VEGF	-0.00	(-0.13, 0.13)	0.98	0.98	0.10	(-0.03, 0.23)	0.20	0.67	0.01	(-0.12, 0.13)	0.94	0.98	-0.00	(-0.13, 0.12)	0.95	0.96
	PlGF	-0.02	(-0.11, 0.07)	0.68	0.91	-0.02	(-0.11, 0.07)	0.67	0.83	0.04	(-0.04, 0.13)	0.42	0.80	-0.08	(-0.17, 0.01)	0.16	0.70
	Angiopoietin	0.26*	(0.01, 0.51)	0.09	0.91	0.35**	(0.10, 0.60)	0.02	0.53	0.23	(-0.01, 0.46)	0.12	0.80	0.18	(-0.07, 0.42)	0.24	0.70
	GCSF	0.06	(-0.01, 0.14)	0.17	0.91	0.10**	(0.03, 0.17)	0.03	0.53	0.02	(-0.05, 0.09)	0.64	0.84	0.01	(-0.06, 0.09)	0.81	0.96
	GM-CSF	-0.07	(-0.21, 0.07)	0.42	0.91	-0.08	(-0.22, 0.06)	0.33	0.72	-0.02	(-0.15, 0.12)	0.82	0.90	-0.23***	(-0.38,-0.09)	0.008	0.28
	IGFBP-3	0.07	(-0.13, 0.26)	0.57	0.91	0.20*	(0.01, 0.39)	0.09	0.63	0.09	(-0.09, 0.27)	0.42	0.80	0.14	(-0.05, 0.34)	0.23	0.70

*** p-value<0.01, ** p-value<0.05, * p-value<0.10

Supplementary Table 4: Changes of the D15 biomarker concentration attributable to randomized intervention, over the standard of care (SOC) group

[1]Results show the change in D15 biomarker concentration associated with the intervention, adjusted for the baseline biomarker level, HIV, sex, site, diarrhoea, WHZ, and oedema. [2]The Benjamini-Hochberg adjusted P-value is included for reference only and has not been used to adjust any significance. P-values were generated using Ancova as described in the methods.

	Budesonide					Teduglutide					N-acetylglucosamine					Colostrum				
Variable	Inf1	Inf2	Inf3	Ent1	Ent2	Inf1	Inf2	Inf3	Ent1	Ent2	Inf1	Inf2	Inf3	Ent1	Ent2	Inf1	Inf2	Inf3	Ent1	Ent2
**HIV**	0.00	-0.19	0.04	-0.30	-0.19	-0.03	0.03	0.05	0.05	0.07	-0.04	0.00	0.04	-0.03	-0.10	-0.02	0.00	0.11	-0.11	-0.03
	(0.98)	(0.07)	(0.77)	(0.02)	(0.18)	(0.76)	(0.79)	(0.68)	(0.69)	(0.63)	(0.68)	(0.99)	(0.72)	(0.85)	(0.51)	(0.85)	(0.99)	(0.38)	(0.42)	(0.86)
**Site**	0.49	-0.15	-0.13	0.03	0.05	0.50	-0.20	-0.05	-0.08	0.05	0.39	-0.13	-0.24	0.04	0.16	0.51	0.07	-0.25	0.15	0.01
	(0.00)	(0.14)	(0.35)	(0.84)	(0.72)	(<0.001)	(0.06)	(0.74)	(0.53)	(0.72)	(0.01)	(0.29)	(0.12)	(0.76)	(0.35)	(<0.001)	(0.55)	(0.08)	(0.30)	(0.93)
**Oedema**	0.27	-0.02	0.24	-0.03	0.04	0.14	-0.08	0.22	-0.18	0.13	0.09	0.04	0.07	0.00	-0.08	0.03	0.10	0.13	0.03	-0.17
	(0.02)	(0.82)	(0.08)	(0.83)	(0.78)	(0.17)	(0.51)	(0.13)	(0.19)	(0.40)	(0.38)	(0.76)	(0.66)	(0.99)	(0.64)	(0.75)	(0.40)	(0.28)	(0.81)	(0.22)
**Diarrhoea**	-0.15	0.08	-0.20	0.19	0.04	-0.06	0.15	-0.02	0.19	-0.10	-0.09	0.04	-0.06	0.04	0.04	0.00	-0.14	0.02	-0.11	0.02
	(0.16)	(0.45)	(0.15)	(0.16)	(0.79)	(0.51)	(0.14)	(0.90)	(0.13)	(0.48)	(0.33)	(0.76)	(0.66)	(0.78)	(0.78)	(0.98)	(0.22)	(0.89)	(0.43)	(0.90)
**Whz**	-0.29	0.06	-0.07	0.37	0.02	-0.26	-0.14	-0.11	0.46	-0.22	-0.23	-0.22	-0.09	0.39	0.21	-0.21	-0.31	-0.03	0.36	0.09
	(0.01)	(0.57)	(0.61)	(0.01)	(0.89)	(0.02)	(0.24)	(0.46)	(0.00)	(0.16)	(0.03)	(0.09)	(0.52)	(0.01)	(0.20)	(0.05)	(0.02)	(0.84)	(0.01)	(0.54)
**Random**	-0.01	-0.13	0.07	-0.04	0.27	0.09	-0.08	0.07	0.04	0.13	0.08	-0.05	0.19	0.03	0.16	0.07	-0.30	0.11	0.18	0.19
	(0.95)	(0.18)	(0.55)	(0.76)	(0.05)	(0.31)	(0.44)	(0.57)	(0.74)	(0.37)	(0.34)	(0.63)	(0.13)	(0.82)	(0.29)	(0.47)	(0.01)	(0.38)	(0.18)	(0.18)
**Inf1(base)**	0.37					0.40					0.52					0.33				
	(0.01)					(0.001)					(<0.001)					(0.004)				
**Inf2(base)**		0.74					0.60					0.74					0.65			
		(<0.001)					(<0.001)					(<0.001)					(<0.001)			
**Inf3(base)**			0.46					0.51					0.41					0.60		
			(0.001)					(0.001)					(0.003)					(<0.001)		
**Ent1(base)**				0.34					0.45					0.45					0.35	
				(0.01)					(0.001)					(0.002)					(0.021)	
**Ent2(base)**					0.46					0.47					0.21					0.50
					(0.002)					(0.002)					(0.20)					(0.001)
**Ent1**	0.21	-0.09	0.30			0.26	0.16	0.24			0.35	0.12	0.37			0.13	0.13	0.19		
	(0.06)	(0.41)	(0.04)			(0.01)	(0.17)	(0.10)			(0.001)	(0.28)	(0.01)			(0.202)	(0.29)	(0.17)		
**Ent2**	-0.13	0.09	-0.02			-0.24	0.40	-0.18			-0.14	0.11	-0.03			-0.34	0.22	0.03		
	(0.22)	(0.38)	(0.85)			(0.01)	(<0.001)	(0.18)			(0.14)	(0.31)	(0.80)			(0.001)	(0.05)	(0.80)		

Supplementary Table 5: Standardised path coefficients from PLS-Path modelling by each randomisation group

Standardised path coefficients with the appropriate p-value from the PLS modelling displayed beneath in brackets.

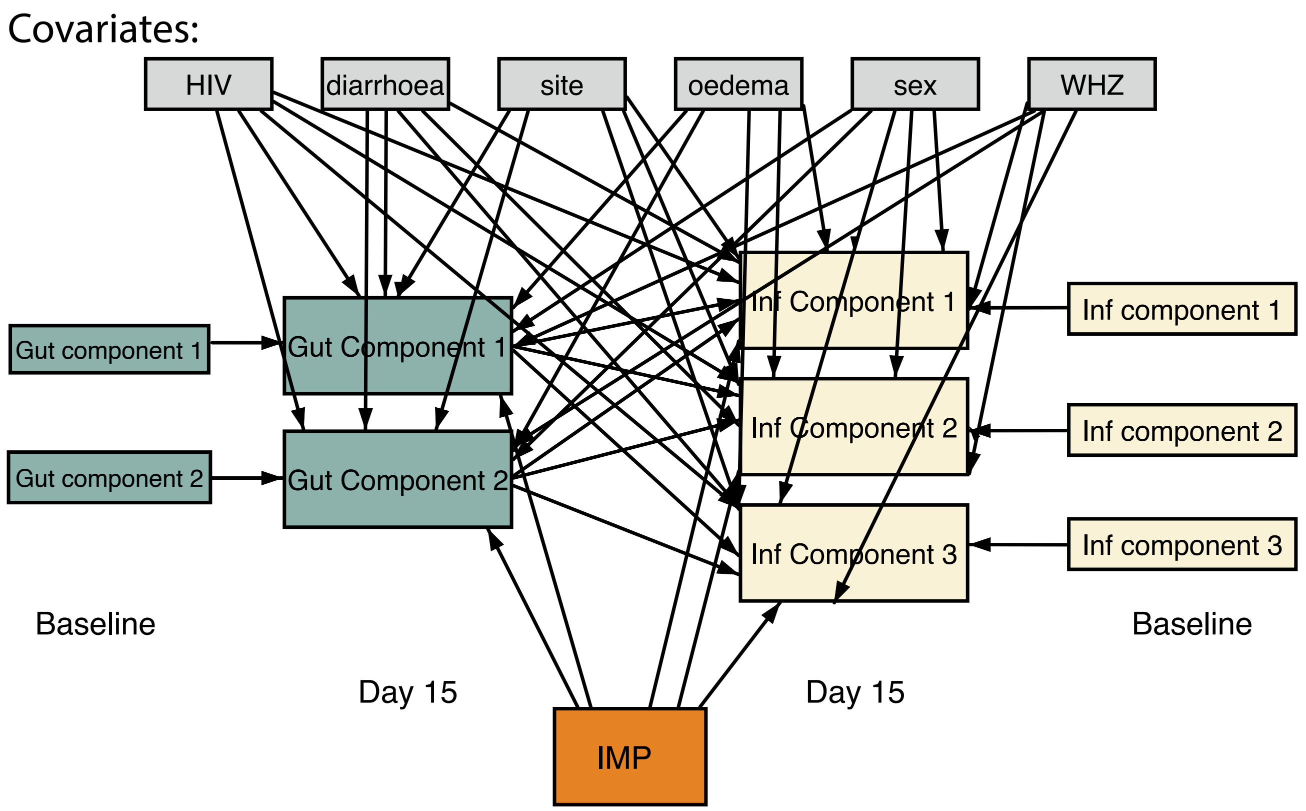

Supplementary Figure 1: Base model tested in structural path modelling

This shows all the base model tested by PLS-path modelling, with the results displayed in Figure 4, and full results shown in Supplementary Table 5. Connections were only shown in Figure 4 if they were significant. All were included in the model regardless of whether they were significant or not. IMP: investigatory medical product, which is the randomization group involved. The groups were tested separately against the standard of care arm.

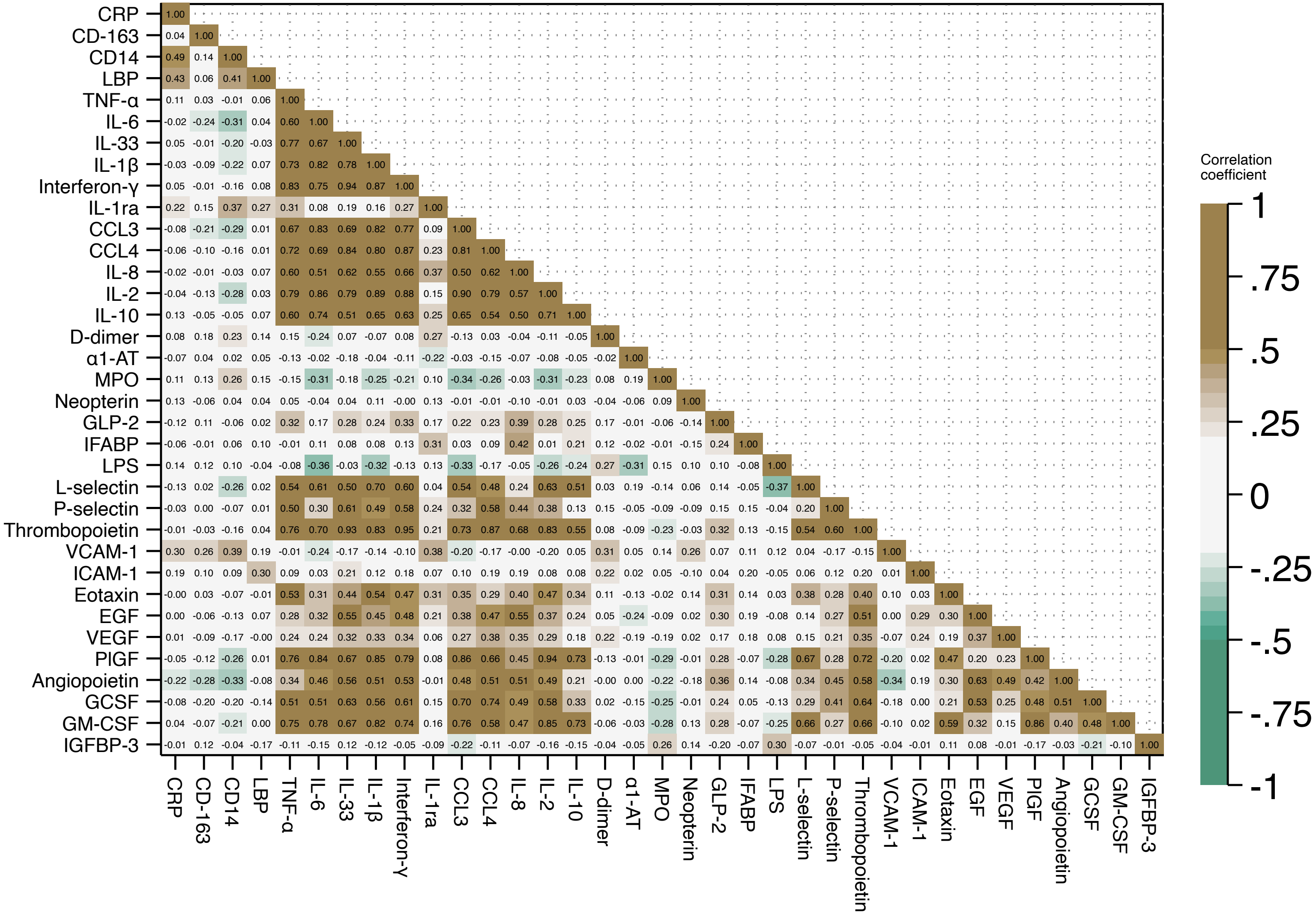

Supplementary Figure 2: Correlation coefficients between the biomarker levels at Day 15

Correlation coefficients between each of the 35 biomarkers concentrations, following normalization by $\log_{10}$ transformation.

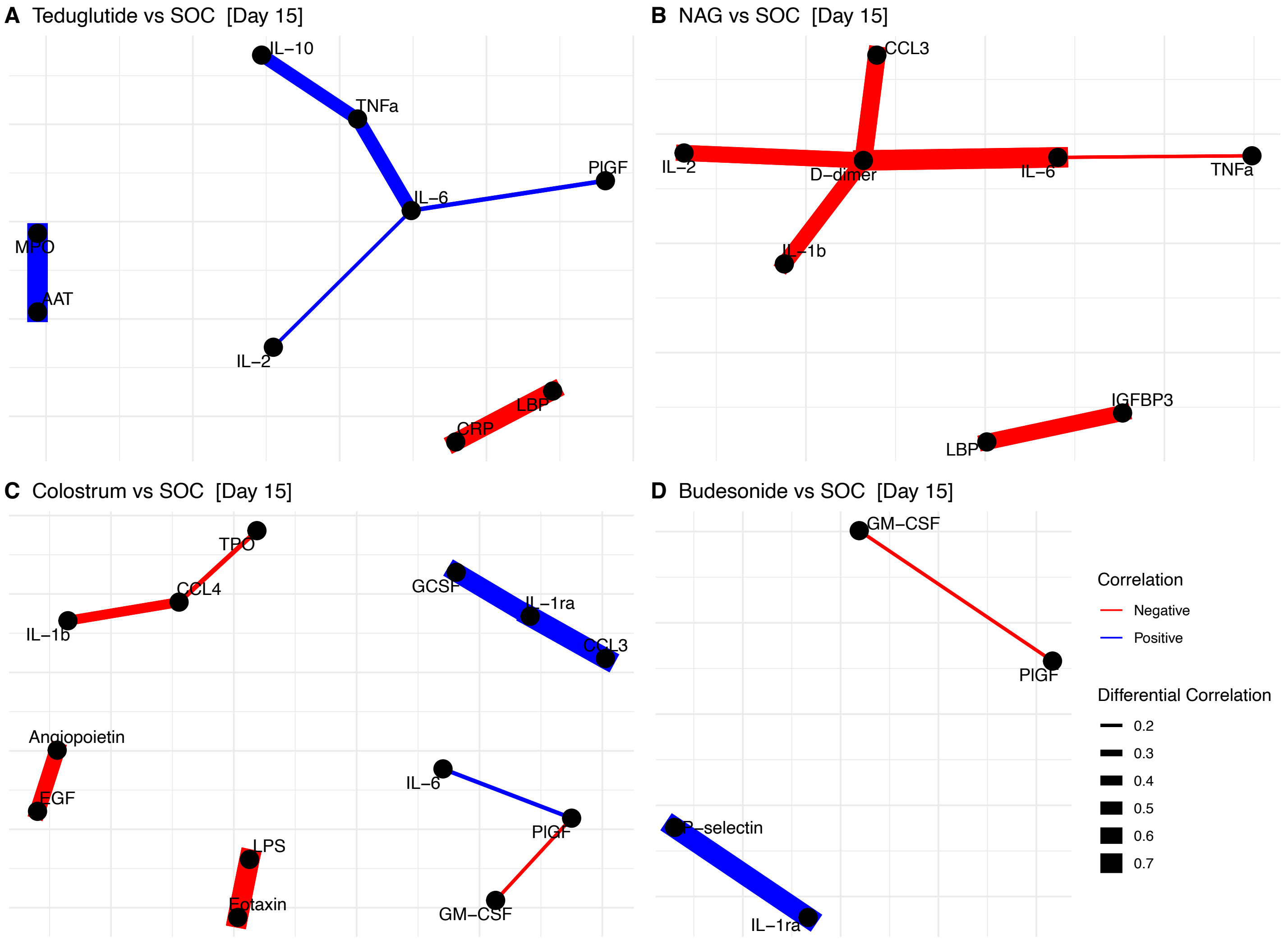

Supplementary Figure 3: Differential correlation network analysis between groups

The correlations between normalized $\log_{10}$ biomarker values at day 15, post-intervention, were compared between A) Teduglutide B) N-acetylglucosamine (NAG), C) colostrum, and D) Budesonide groups and the standard of care (SOC) using the Fisher's Z transformation of the Pearson correlation coefficient in each group, with significance determined by Fisher's Z test. The threshold of significance was P<0.10, with false discovery rate correction.

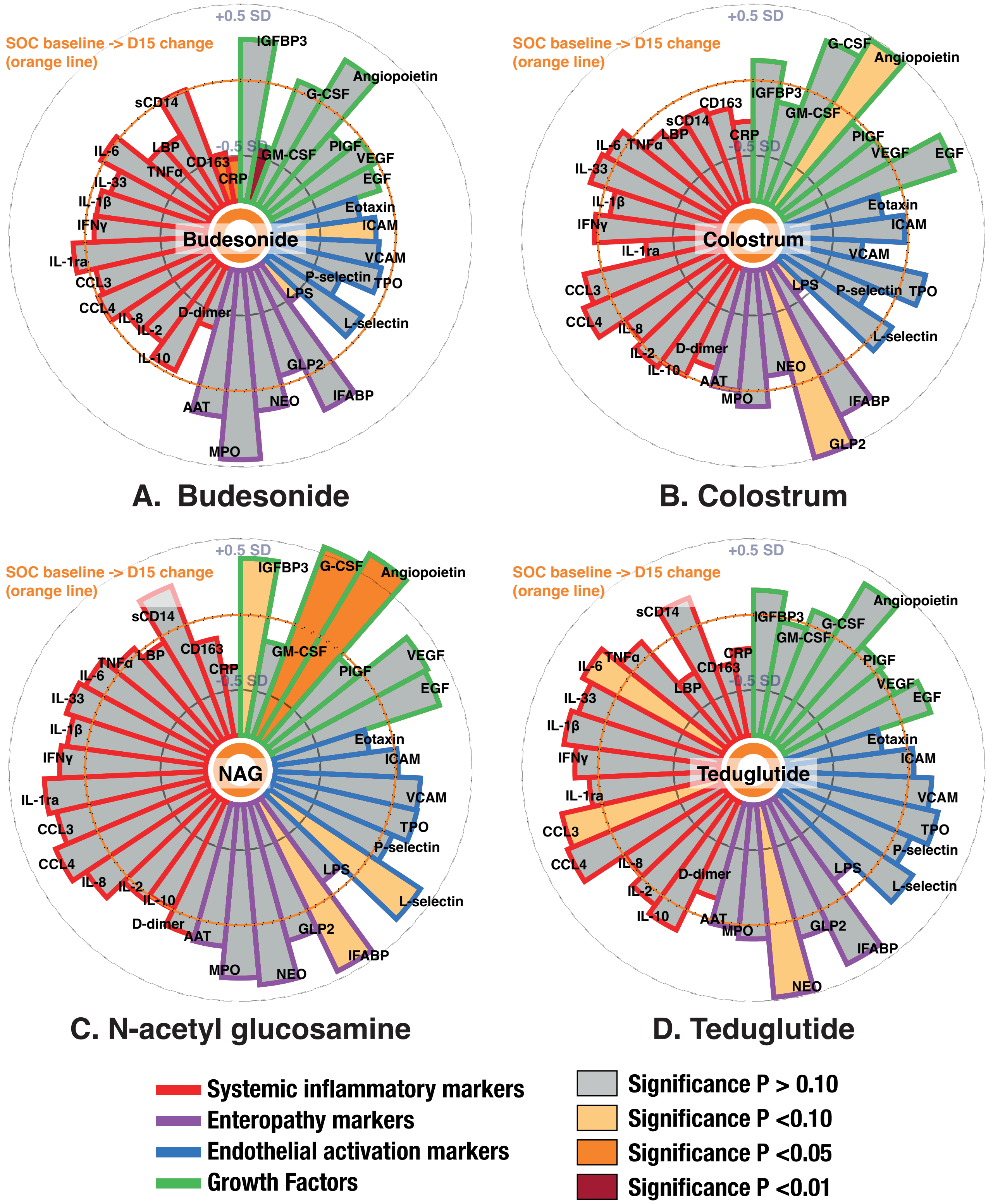

Supplementary Figure 4: Changes of the adjusted log10 D15 biomarker value attributable to randomized intervention, over the standard of care (SOC) group

Differences are shown in A) budesonide B) colostrum C) N-acetylglucosamine (NAG) and D) teduglutide groups. Results were adjusted for sex, oedema, HIV, diarrhoea, WHZ, site, and the baseline biomarker value. Each biomarker has its own assigned ray, and the length of the ray shows how the result differs in comparison to the SOC group, which is shown as the level of the labelled orange circle. If the ray is outside the orange circle, the biomarker value in this treatment group is higher than in the SOC group; if the ray is inside the orange circle the biomarker level in this treatment group is lower than in the SOC group. The ray is coloured according to the p value from the multivariable model. A p value threshold <0.10 from ancova modelling was pre-specified as statistically significant since this is a phase II trial.The full numerical results are shown in Supplementary Table 4.

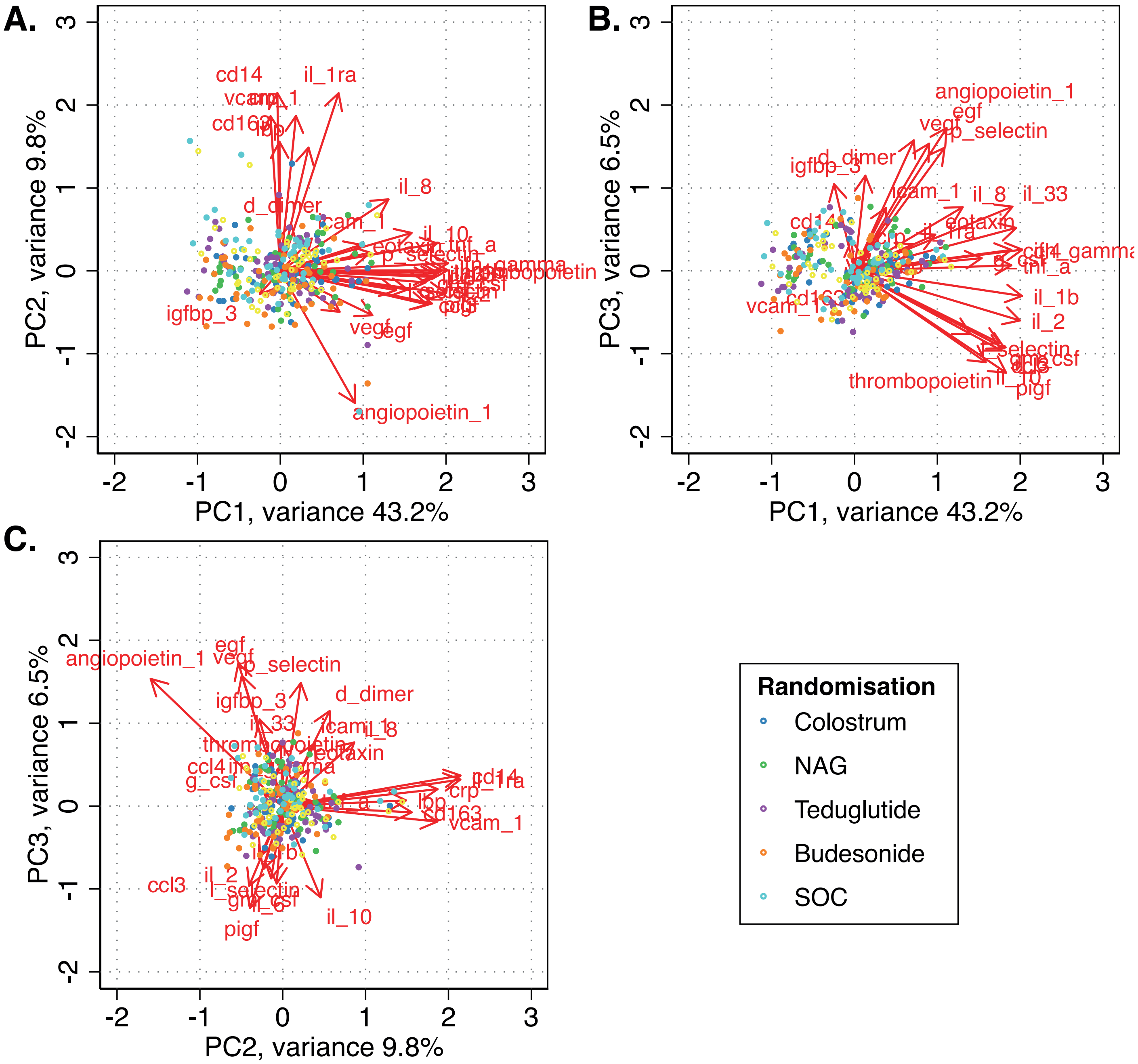

Supplementary Figure 5: Principal component analysis plots of the all-timepoint results showing the individual participants split by randomization

This shows A) PC2 against PC1; B) PC3 against PC1; C) PC3 against PC2; the colours of the dots represent the randomization arms.

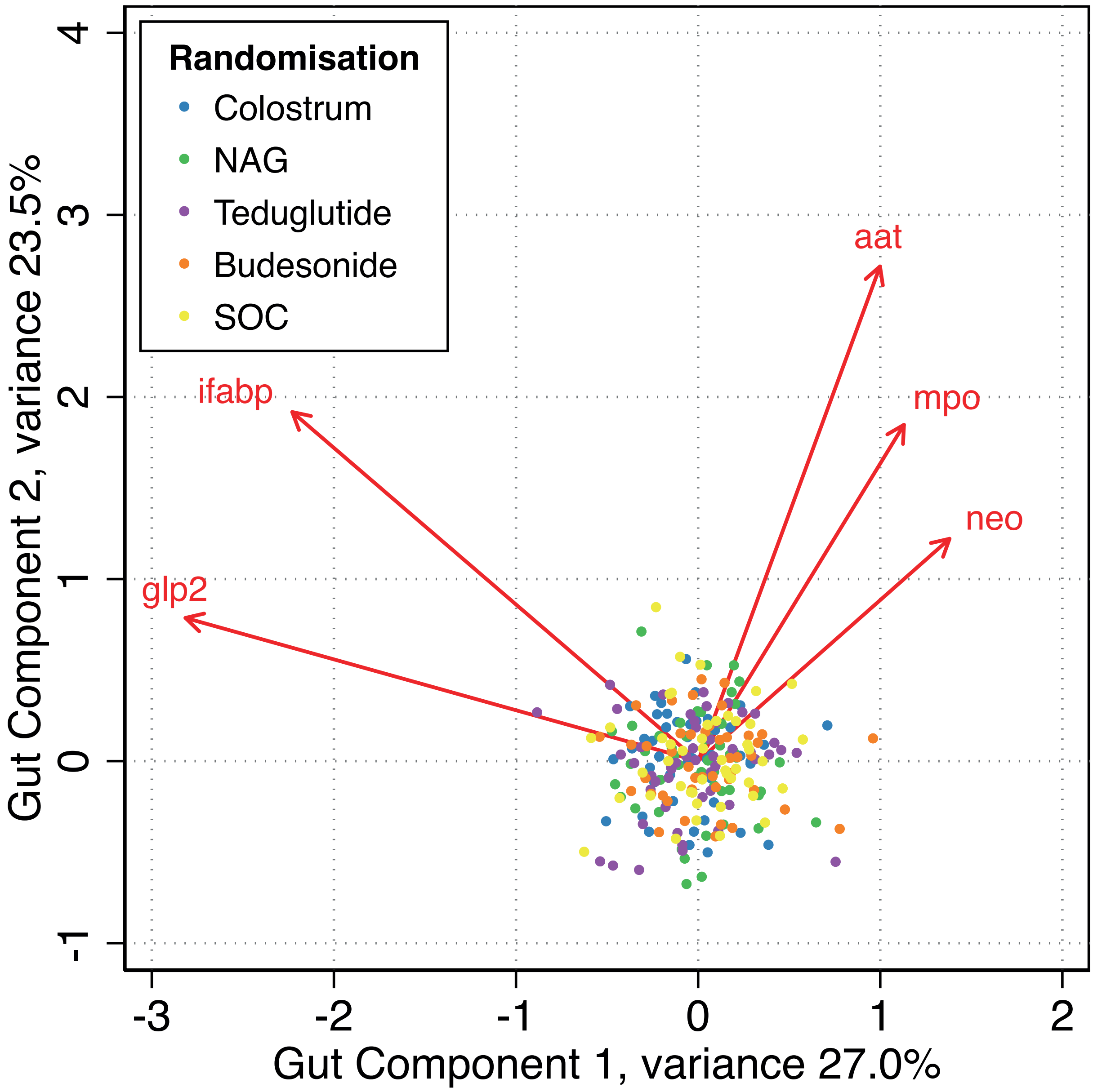

Supplementary Figure 6: PCA plots for the gut components at all timepoints

This shows A) Gut Component 2 against Gut Component 1; and B) the screeplot for the PCA analysis of gut biomarkers
